# Investigation of Deconvolution Method with Adaptive Point Spread Function Based on Scintillator Thickness in Wavelet Domain

**DOI:** 10.3390/bioengineering11040330

**Published:** 2024-03-28

**Authors:** Kyuseok Kim, Bo Kyung Cha, Hyun-Woo Jeong, Youngjin Lee

**Affiliations:** 1 Department of Biomedical Engineering, Eulji University, 553, Sanseong-daero, Sujeong-gu, Seongnam-si 13135, Republic of Korea; kskim502@eulji.ac.kr; 2 Precision Medical Device Research Center, Korea Electrotechnology Research Institute (KERI), 111 Hanggaul-ro, Sangnok-gu, Ansan-si 15588, Republic of Korea; bkcha@keri.re.kr; 3 Department of Radiological Science, Gachon University, Incheon 21936, Republic of Korea

**Keywords:** image deblurring, adaptive point spread function (PSF), wavelet transform, image quality assessment (IQA), radiography

## Abstract

In recent years, indirect digital radiography detectors have been actively studied to improve radiographic image performance with low radiation exposure. This study aimed to achieve low-dose radiation imaging with a thick scintillation detector while simultaneously obtaining the resolution of a thin scintillation detector. The proposed method was used to predict the optimal point spread function (PSF) between thin and thick scintillation detectors by considering image quality assessment (IQA). The process of identifying the optimal PSF was performed on each sub-band in the wavelet domain to improve restoration accuracy. In the experiments, the edge preservation index (EPI) values of the non-blind deblurred image with a blurring sigma of σ = 5.13 pixels and the image obtained with optimal parameters from the thick scintillator using the proposed method were approximately 0.62 and 0.76, respectively. The coefficient of variation (COV) values for the two images were approximately 1.02 and 0.63, respectively. The proposed method was validated through simulations and experimental results, and its viability is expected to be verified on various radiological imaging systems.

## 1. Introduction

X-ray-based imaging is widely used in industry and medicine [1,2,3]. X-ray images that are acquired using film and intensifier systems have enabled the development of computed radiography (CR) imaging technology and digitization. CR has the advantage of being able to display X-ray digital images in multiple locations and is applied in software technologies. However, CR has a disadvantage in that the imaging plate must be erased to enable reuse, and various traces may appear in the resulting X-ray image. The digital radiography (DR) X-ray imaging system was developed as a method for maintaining the advantages of CR while addressing the disadvantages. As such, DR is a popular technology that is used in various fields [4].

In the acquisition of X-ray images using DR imaging systems, the basic components of the source and detector play an essential role. Among the components used for acquiring X-ray images, the image quality changes significantly based on the type and characteristics of the detector. The flat panel type is widely used as a detector in DR X-ray imaging systems [5]. Flat-panel detectors (FPDs) are broadly classified into direct and indirect conversion types based on the presence or absence of a light conversion process [6]. Direct-conversion-type X-ray detectors, which are used in combination with an active matrix array, mainly use amorphous selenium (a-Se) materials. In indirect X-ray detectors, the technology for manufacturing an amorphous silicon (a:Si) photodiode on a thin-film transistor (TFT) array is used, thereby attaching the array to a scintillator. Although X-ray images obtained with a direct-conversion-type detector have superior spatial resolution compared to those obtained with indirect conversion, they have the disadvantage of having low detection sensitivity [7,8]. Therefore, indirect conversion-type X-ray detectors can be safely used at relatively low doses in the medical field. According to Fischbach’s study, the indirect FPD system outperforms the direct conversion method in terms of image quality and receiver operating characteristics when the dose is reduced [7].

Sensitivity can be improved by increasing the thickness of the indirect conversion-type X-ray detector. However, thick X-ray detectors result in a decrease in spatial resolution, and a method is required to address this [9]. Thus, the deconvolution method has been introduced as an approach for improving resolution. Classical deconvolution methods, including inverse filtering and Wiener filtering [10,11,12,13], have been widely used in the field. This approach provides fast results but has limitations in terms of image quality because of noise amplification. Several prior-based deconvolution methods have been proposed to address these issues. Gaussian and Laplacian priors are representative priors that effectively improve resolution but produce excessive blurring and ringing artifacts [14,15]. Lucy–Richardson deconvolution and bilateral filtering reduce the ringing artifact but require numerous parameters [16,17]. The half-quadratic minimization approach effectively deblurs using total variation regularization with priors [15,18]. In addition to effective deconvolution, the most important aspect of non-blind deconvolution is identifying the correct blur information. A non-blind deconvolution method that performs deconvolution by measuring the point spread function (PSF) or line spread function (LSF) after radiation exposure, using a pinhole (Ø~10 μm), slit, or edge phantom under radiation quality (RQA) conditions, was proposed based on the IEC standard for general radiography (IEC 62220-1-1:2015 International Standard) [19,20]. This is the most common method for measuring degradation in radiographic imaging systems; however, applying it to portable X-ray systems that function under a variety of exposure conditions is difficult. Another limitation is that it does not consider noise. 

Recently, a study was conducted to convert the sharpness of an image in the thick scintillator into a thin scintillator detector image considering the noise characteristics in the thick scintillator detector image [21]. The structural similarity index (SSIM) [22] and feature similarity index (FSIM) [23] were used to derive the optimal size of the blur kernel considering the entire image characteristics, and a deconvolution was performed based on this to confirm the improved results. However, there is a limit to detailed image restoration because of the image-wide properties of noise. Because the noise characteristics of low and high frequencies are different, various approaches have studied restoration through domain transformation [24,25]. In particular, wavelet-based image restoration is known to enable detailed image restoration by analyzing the image quality characteristics of each sub-band. We present a method for deriving an optimal parameter for effective image restoration in the existing method based on wavelet transformation [21], thereby confirming that the proposed method can contribute to the effective image restoration of radiographic images. 

This study aimed to improve image performance by considering the relationship between thin- and thick-thickness scintillation detectors using the wavelet transform. This concept involves using a thick scintillation detector for low-dose radiation exposure, and the proposed algorithm is used to achieve the resolution of a thin scintillation detector. In the process of identifying the optimal degradation information between the two detectors, the optimal kernel is predicted based on image quality assessment (IQA) in each sub-band region following the wavelet transform for precise analysis. In the following sections, the implementation of the proposed algorithm is briefly described, and the simulation and experimental results are presented.

## 2. Materials and Methods

### 2.1. Proposed Optimal Deblurring Framework

The proposed algorithm introduces the IQA perspective to overcome the limitations of the traditional deblurring method, which focuses primarily on improvements in absolute quantitative evaluation factors. The proposed algorithm uses SSIM and gradient of magnitude (GM) [26], which are well-known evaluation factors in IQA, to derive an optimal PSF for restoring the sharpness of an image from a thick scintillation detector to that of a thin scintillation detector. The SSIM and GM factors can be defined using Equations (1) and (2) as follows:

(1) $$SSIM=\frac{\left(2\mu_{x}\mu_{y}+C_{1}\right)\left(2\sigma_{xy}+C_{2}\right)}{\left(\mu_{x}^{2}+\mu_{y}^{2}+C_{1}\right)\left(\sigma_{x}^{2}+\sigma_{y}^{2}+C_{2}\right)},C_{1}={\left(k_{1}L\right)}^{2},C_{2}={\left(k_{2}L\right)}^{2},$$

(2) $$GM=\sqrt{{\left(I⨂f_{h}\right)}^{2}+{\left(I⨂f_{v}\right)}^{2}},$$

where u and σ denote the local mean and the standard deviation, respectively. Here, $\sigma_{xy}$ represents the cross-covariance of images x and y; C represents a positive constant (e.g., k_1_ and k_2_ are 0.01 and 0.03 in this study, respectively); and L denotes the dynamic range. Values close to one indicate that the reference and comparison images are similar in terms of luminance, contrast, and structure. $f_{h}$ and $f_{v}$ denote the gradient operators in the horizontal and vertical directions, respectively. The gradient operator can be calculated by adopting methods such as Sobel, Prewitt, Laplacian, and Canny [27]. ⨂ is the convolution operator and I is the input image. The GM value represents the sharpness of the image. The GM value of an image acquired using a thin scintillation detector was used as a reference. In the proposed algorithm, the noise component has a significant impact on image performance. Therefore, the discrete wavelet transform (DWT) [28], which is a representative method in multi-resolution analysis (MRA), was used to predict a suitable PSF considering the characteristics of the noise component as follows:

(3) $$\varphi_{j,k}\left(x\right)=2^{\frac{j}{2}}\varphi \left(2^{j}x-k\right),\psi_{j,k}\left(x\right)=2^{\frac{j}{2}}\psi \left(2^{j}x-k\right),$$

where $\varphi_{j,k}\left(x\right)$ denotes the scaling function considering the dilation factor j and translation factor k in input information x. The DWT was computed to discriminate based on the orthonormal basis of a class function, thereby reconstructing the inverse DWT from a low-pass filtered (LPF) image and approximate coefficients of the decomposed data [29]. The input image was separated into low- and high-frequency sub-band images to determine the optimal PSFs.

Algorithm 1 outlines the adaptive PSF prediction in each sub-band image. Briefly, the acquired thin scintillation detector image (*IMG*_1_) and thick scintillation detector image (*IMG*_2_) are input into the proposed algorithm to perform DWT. A single level of DWT is performed in this study to obtain sub-band images of low (*IMG_L*) and high frequencies in the horizontal, vertical, and diagonal directions (*IMG_H*, *IMG_V*, and *IMG_D*). The PSFs are then generated, using Equations (1) and (2) based on the sigma size and convolution with the sub-band images of *IMG_1_* to create blurred images (*IMG_1__L_blur_*, *IMG_1__H_blur_*, *IMG_1__V_blur_*, and *IMG_1__D_blur_*). *IMG_1__L_blur_* is compared with *IMG_2__L* using Equations (1) and (2), and the results are stacked in the *SSIM_val_* matrix. The other sub-band images are computed according to Equation (2) and stored in their resultant matrices (*GM_H_val_*, *GM_V_val_*, and *GM_D_val_*). At the end of the loop, the sigma value of the largest *SSIM_val_* matrix is selected and adopted as the *PSF_L_* value. The *PSF_H_*, *PSF_V_*, and *PSF_D_* values were the most similar sigma values between the GM metrics of the high-frequency sub-band images of *IMG*_1_ and GM values of *IMG*_2_.

The low-resolution image of the thick scintillation detector was restored to its optimal resolution using the optimal PSFs (*PSF_L_*, *PSF_H_*, *PSF_V_*, and *PSF_D_*). As a non-blind deconvolution method, we used the *l*_1_-norm-based total variation (TV) minimization in Equation (4), which was shown to be effective in terms of artifact suppression in previous studies [30,31].

(4) $$f^{*}=\underset{{\mathrm{IMG}}_{2\_\mathrm{sub}}\in Q}{\mathrm{argmin}}{\left\|{PSF}_{sub}⨂⨂{IMG}_{2\_sub}-{IMG}_{2\_sub}\right\|}_{2}^{2}+\lambda {\left\|\nabla {IMG}_{2\_sub}\right\|}_{1},$$

where Q denotes the set of feasible ${IMG}_{2\_sub}$, containing the sub-band images after DWT of *IMG_2_*; ${PSF}_{sub}$ is an adaptive PSF scheme based on the ${IMG}_{2\_sub}$; and λ is a constant for balancing the fidelity and regularization terms and was empirically set to 0.01. Finally, the restored image is obtained when the mismatch between the updated images is lower than a certain tolerance value (i.e., a tolerance of 1 × 10^−4^ was used in the study). This solution uses the augmented Lagrangian method [32]. The final image is obtained by performing an inverse DWT on each sub-band-restored image.
**Algorithm 1** Structure of proposed algorithm framework for predicting optimal PSF**1:      Input**: Initial 2D matrix *IMG_1_, IMG_2_* **2:**      **Output:** Complete 2D matrix *PSF_L_, PSF_H_, PSF_V_, PSF_D_*
**3:**      **Function**
*Initialize* ():
**4:**            *Sigma_val_* = 0.01 to 4 (empirically);
**5:**            Preallocation (*SSIM_val_*, *GM_H_val_*, *GM_V_val_*, *GM_D_val_*);
**6:**      **END**
**7:**      **Function**
*Main* ():
**8:**              *IMG_1__L, IMG_1__H, IMG_1__V, IMG_1__D*
← DWT (*IMG_1_*);
**9:**              *IMG_2__L, IMG_2__H, IMG_2__V, IMG_2__D*
← DWT (*IMG_2_*);
**10:**            **For**
*val* = *Sigma_val_* (start): *Sigma_val_* (end) **do**
**11:**                         *PSF_val_* ← Input sigma according to the *val*;
**12:**                        
*IMG_1__L_blur_* = *IMG_1__L* ⨂⨂ *PSF_val_*;
**13:**                        *IMG_1__H_blur_* = *IMG_1__H* ⨂⨂ *PSF_val_*;
**14:**                        *IMG_1__V_blur_* = *IMG_1__V* ⨂⨂ *PSF_val_*;
**15:**                        *IMG_1__D_blur_* = *IMG_1__D* ⨂⨂ *PSF_val_*;
**16:**                        *SSIM_val_* (val) ← Calculate Equation (1) (*IMG_1__L_blur_*, *IMG_2__L*);
**17:**                        *GM_H_val_* (val) ← Calculate Equation (2) (*IMG_1__H_blur_*);
**18:**                        *GM_V_val_* (val) ← Calculate Equation (2) (*IMG_1__V_blur_*);
**19:**                        *GM_D_val_* (val) ← Calculate Equation (2) (*IMG_1__D_blur_*);
**20:          END For**

**21:**          *PSF_L_* = find the index *Sigma_val_* (max (*SSIM_val_*));
**22:**          *GM_H_Ref_*
← Calculate Equation (2) (*IMG_2__H*);
**23:**          *GM_V_Ref_*
← Calculate Equation (2) (*IMG_2__V*);
**24:**          *GM_D_Ref_*
← Calculate Equation (2) (*IMG_2__D*);
**25:**          *PSF_H_* = find the index *Sigma_val_* (*GM_H_val_* ~= *GM_H_Ref_*);
**26:**          *PSF_V_* = find the index *Sigma_val_* (*GM_V_val_* ~= *GM_V_Ref_*);
**27:**          *PSF_D_* = find the index *Sigma_val_* (*GM_D_val_* ~= *GM_D_Ref_*);
**28:    Return**
*PSF_L_, PSF_H_, PSF_V_, PSF_D_*
**29:    END**

### 2.2. Simulation and Experiment Conditions

The image degradation model [33] defined in Equation (5) was used to conduct the simulations:

(5) $$g\left(x\right)=PSF\left(x\right)⨂f\left(x\right)+n\left(x\right),PSF\left(x\right)=\exp\left(-\frac{{\left(x-\mu \right)}^{2}}{2\sigma^{2}}\right),$$

where g denotes the degraded image with a blur component obtained by convolving the PSF and clean image (f), and the noise component (n) is obtained by adding Poisson and Gaussian noise; x denotes the discrete index coordinate, and μ and σ denote the center position and standard deviation of PSF, respectively. Here, the PSF was assumed to follow a Gaussian distribution; the size of the PSF was 31 × 31. The projection image (f) was obtained through the ray-tracing technique, and the X-ray exposure conditions were 70 kV and 5 mAs with a source-to-detector distance (SDD) of 1500 mm and a source-to-object distance (SOD) of 300 mm. An additional filter was used to reduce the energy to 21.0 mmAl. The σ of thin and thick scintillators was measured as approximately 2.61 and 5.13 pixels, respectively [19,20]. A noisy image was generated in accordance with the Poisson and Gaussian noise parameters. We used the *imnoise* (·) and *poissrnd* (·) functions to design the noisy image from the blurred image in the MATLAB toolbox (R2021a, MathWorks, Natick, MA, USA).

**Figure 1:**
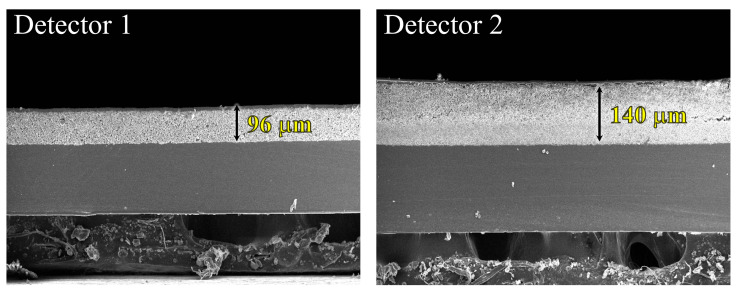
Scanning electron microscope (SEM) images of indirect conversion detectors; thin-thickness scintillation of 96 μm and thick-thickness scintillation of 140 μm.

In the experiments, the radiographic exposure conditions were the same as those in the simulation. Figure 1 shows scanning electron microscopy (SEM) images reflecting the scintillator thickness of each detector. The thickness of the thin scintillator (detector 1) was 96 μm, and that of the thick scintillator (detector 2) was 140 μm. Each detector used a Gd_2_O_2_S:Tb scintillator and a complementary metal-oxide-semiconductor (CMOS) image sensor. The flat panel detector had a pixel size of 48 μm, pixel matrix of 512 × 1024 pixels, and analog-to-digital conversion resolution of 16 bits.

**Figure 2:**
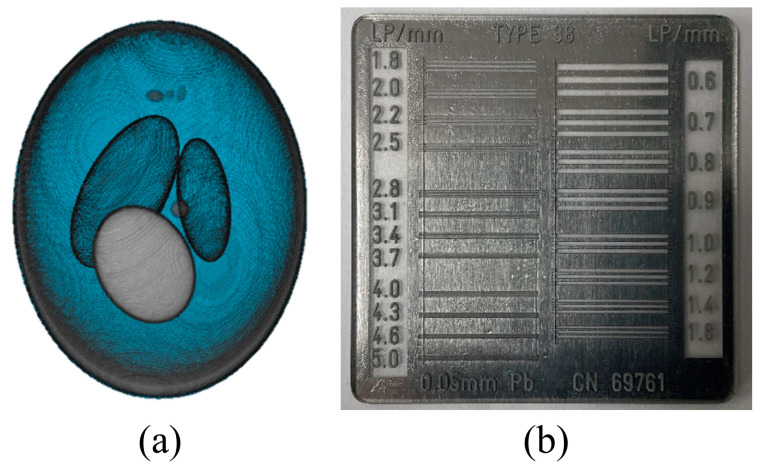
Examples of (**a**) 3D numerically modified Shepp–Logan phantom used in the simulation and (**b**) high-resolution line chart phantom (Type 38, CN 69761, Active Radsys, Italy) used in the experiments.

Figure 2 shows the (a) 3D numerically modified Shepp–Logan phantom used for the simulations and (b) high-resolution line chart phantom (Type 38, CN 69761, Active Radsys, Ravenna, Italy) used for the experiments. The numerical phantom had 300 × 300 × 300 voxels and a 50 μm voxel size. The line chart phantom comprised 20 groups ranging from 0.6 to 5.0 lp/mm. Line-group tests demonstrate the effectiveness of these phantoms in assessing visual resolution values. Depending on the system under evaluation, these tests are available across a range of spatial frequencies and increments. Their primary benefit is their ability to reliably avoid reading errors caused by pseudo-sharpness. This is advantageous in quantitative performance evaluations before performing clinical validation.

The proposed scheme was implemented on a normal workstation (OS: Windows 10, CPU: 2.13 GHz, and RAM: 64 GB).

### 2.3. Quantitative Evaluation for Image Quality

To evaluate the image performance, the spatial resolution and noise characteristics were evaluated using the edge preservation index (EPI) [34] and coefficient of variation (COV) [35]. The EPI is a metric used to formulate edge preservation that is robust to noise and is highly correlated with the human vision system. The EPI is calculated as follows:

(6) $$EPI=\frac{\Gamma \left(⊿p_{1}-\bar{⊿p_{1}},⊿p_{2}-\bar{⊿p_{2}}\right)}{\sqrt{\left(⊿p_{1}-\bar{⊿p_{1}},⊿p_{1}-\bar{⊿p_{1}}\right)}\circ \Gamma \left(⊿p_{2}-\bar{⊿p_{2}},⊿p_{2}-\bar{⊿p_{2}}\right)},\Gamma \left(a,b\right)=\sum_{t\in ROI}^{T}a\left(t\right)b{\left(t\right)}^{2},$$

where ⊿ denotes the gradient of the reference image ($p_{1}$) and measured image ($p_{2}$); $\bar{⊿p}$ denotes the implementation of Laplacian filtering in the region of interest (ROI); and T is the total pixel number of the image. The closer the EPI value is to one, the more edge properties are preserved in the image data. The COV factor and relative variation allow quantitative analysis by normalizing the variation, as expressed in Equation (7), as follows:

(7) $$COV=\frac{SD}{Mean},$$

where SD and mean denote the standard deviation and mean value of the target image, respectively. In general, a lower COV value indicates that the variation rate is relatively small because of the characteristics of noise and is expected to clearly identify objects.

## 3. Results and Discussion

**Figure 3:**
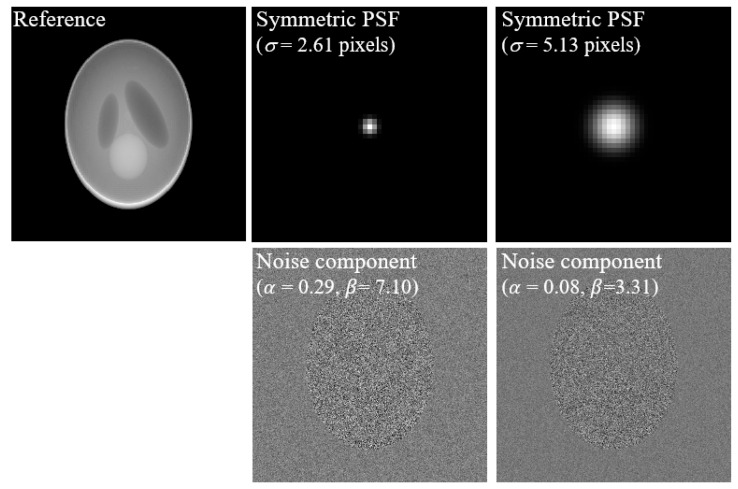
Example of a clean image as reference; symmetric PSFs (e.g., σ = 2.61 pixels of detector 1 and σ = 5.13 pixels of detector 2); and images with noise parameters (e.g., α = 0.29, β = 7.10 of detector 1, and α = 0.08, β = 3.31 of detector 2).

Figure 3 shows a clean projection image of the reference 3D Shepp–Logan phantom (top left). We measured the modulation transfer function (MTF) using an edge phantom on an actual imaging system [19,20] using detectors 1 and 2, thereby determining that the sigma of the PSF of detector 1 was 2.61 pixels and that of detector 2 was 5.13 pixels. The symmetric PSF shown in Figure 3 was obtained by applying Equations (2)–(5) and using the measured sigma values. In addition, the noise level of detectors 1 and 2 was estimated using the non-parametric detection method of Sutour et al. [36]. This method selects homogeneous patches from the original image and estimates a first-order equation with Poisson noise as the slope (α) and Gaussian noise as the y-intercept ($\beta^{2}$) based on the mean and variance of each patch. The α of detectors 1 and 2 was approximately 0.29 and 0.08, respectively. The β of the two detectors was approximately 7.10 and 3.31, respectively. According to each parameter, each noise image was generated using the *imnoise* (·) and *poissrnd* (·) functions, and the final noise component image was designed by adding them.

**Figure 4:**
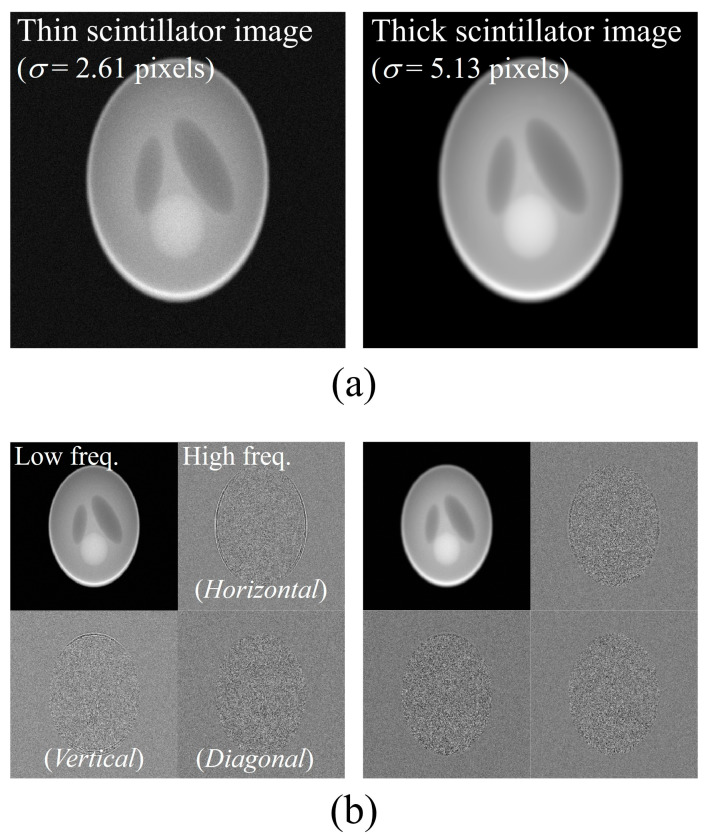
(**a**) Degraded images of thin scintillation (**left**) and thick scintillation (**right**) detectors using the PSF and noise parameters in the simulation; (**b**) results of the wavelet transform.

Figure 4a shows degradation images of the thin (left) and thick scintillation detectors (right) using the PSF and noise parameters shown in Figure 3 in the simulations. Qualitatively, the simulation image from the thin scintillation detector contains less blur and more noise components than those from the thick scintillation detector. This implies that the simulation verification can be considered adequate for validating the proposed algorithm. Figure 4b shows the wavelet transform results at only one level. In this study, the proposed method based on sub-band images separated using the DWT was used to determine the optimal PSF for each image. In wavelet transformation, the high-frequency region is applied in horizontal, vertical, and diagonal directions depending on the differential direction. Different noise characteristics for each sub-band can be confirmed. Only one level of DWT was performed because we did not acquire significant insight into the results from the image restored using the proposed method after separating it into numerous levels.

**Figure 5:**
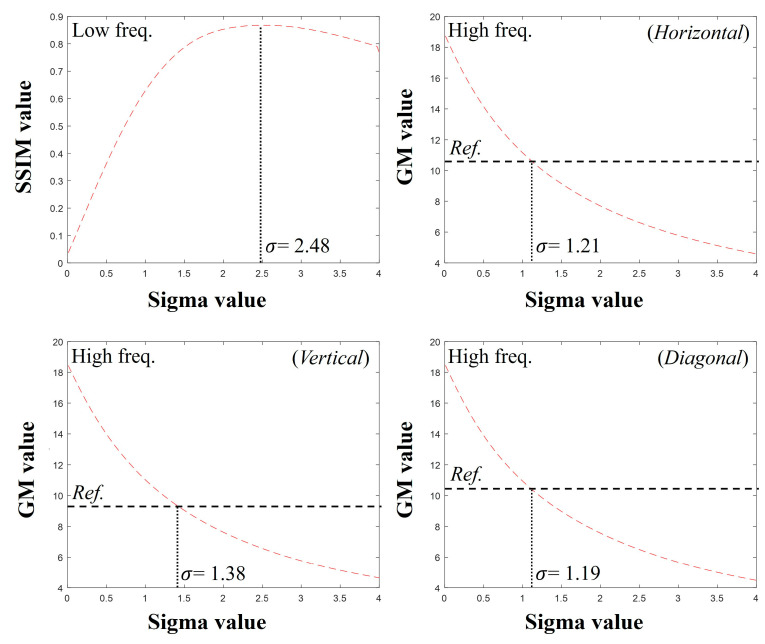
Graphs of SSIM and GM values using the proposed method for detectors 1 and 2. The SSIM graph uses a low-frequency sub-band image, and the other graphs use high-frequency images.

**Figure 6:**
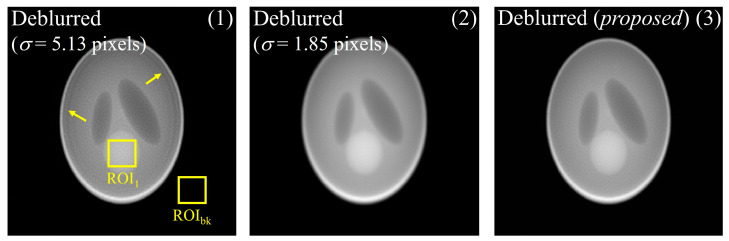
Results of images deblurred using σ = 5.13 pixels and σ = 1.85 pixels without DWT and proposed image. Here, yellow arrows are an artifacts caused by deburring using oversized PSF.

Figure 5 shows the plots of the SSIM and GM values obtained by comparing the images of detector 1 after convolving various PSFs with detector 2. The SSIM comparison between the low-frequency sub-band images from the DWT showed the highest score at σ = 2.48 pixels. For the high-frequency sub-band images in the horizontal, vertical, and diagonal directions of the DWT, the GM values were most consistent with those of the reference data obtained from detector 2 at σ = 1.21 pixels, σ = 1.38 pixels, and σ = 1.19 pixels, respectively. Figure 6 shows the results of the image restoration of detector 2 based on these parameters.

**Figure 7:**
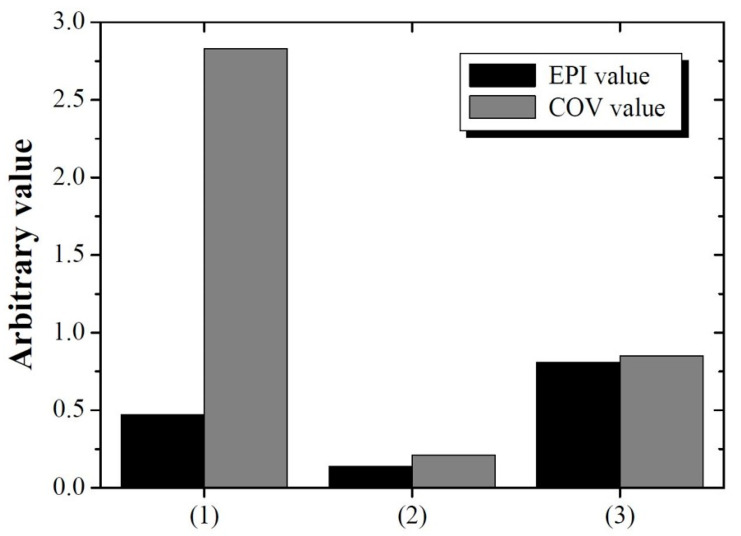
EPI and COV values of the three images in Figure 6. Here, the COV factor is the average value of the COVs calculated in the background (ROI_bk_) and target (ROI_1_) areas.

In comparisons of (1) the image with deconvolution performed using σ = 5.13 pixels for detector 2, (2) the image on which deblurring was performed using σ = 1.85 pixels, and (3) the image yielding the highest SSIM value between detectors 1 and 2 without DWT, the image obtained with the proposed method exhibited qualitatively higher sharpness than that without DWT. In the case of deblurring based on the system degradation information of detector 2 (σ = 5.13 pixels), the result of deblurring improved the sharpness compared with that of the proposed method. However, problems such as artifact generation in yellow arrows and noise amplification did occur. This can be the result of attempting to improve the sharpness of the edge from the noisy image, which has a negative impact on image quality. This tendency was also reflected in the quantitative evaluation shown in Figure 7. The EPI values of images (1), (2), and (3) were approximately 0.47, 0.14, and 0.81, respectively. This comparison used the image from detector 1 as a reference. The EPI of the image in (1) was lower than that of the proposed image in (3) and is expected to reflect the distortion information caused by artifacts. The COV values of the three images were approximately 2.83, 0.21, and 0.85, respectively. A comprehensive review of these results demonstrates that the proposed method considers sharpness and noise when performing image restoration, which is akin to human perception. Furthermore, we confirmed that the optimal PSF derivation by MRA using DWT can predict the PSF more precisely than the PSF derived from only the image domain.

**Figure 8:**
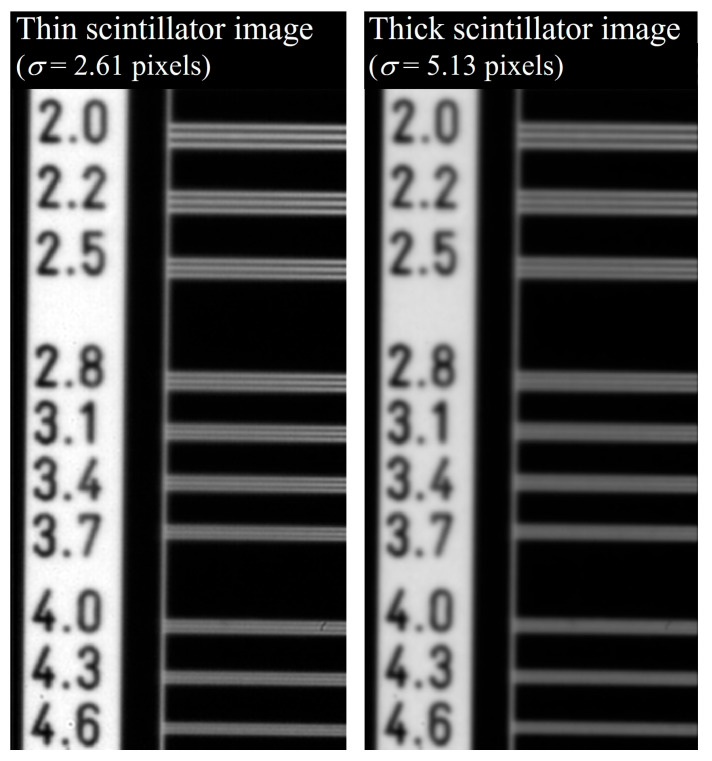
Example of thin scintillator image with σ = 2.61 pixels and thick scintillator image with σ = 5.13 pixels using the high-resolution line chart phantom in Figure 2b.

**Figure 9:**
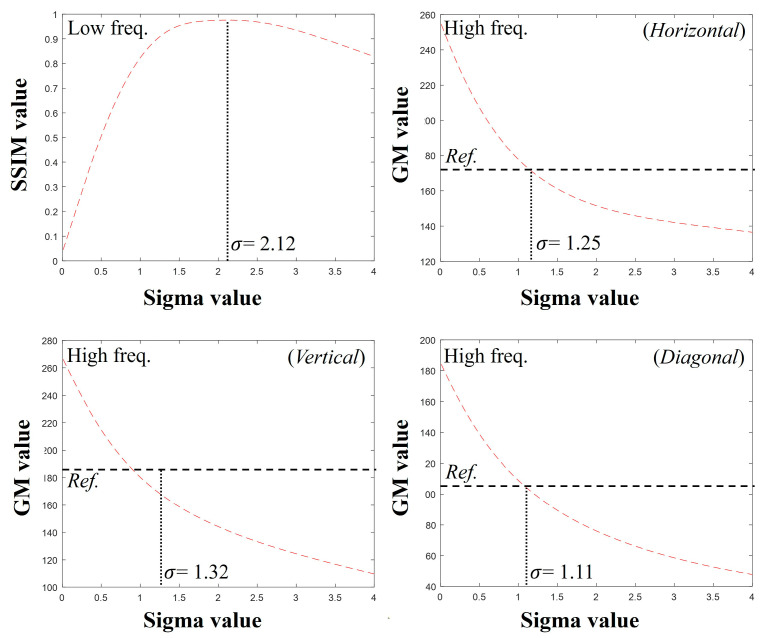
Plots of predicted optimal sigma values in DWT sub-band images using SSIM and GM factors.

Figure 8 shows radiographic images of the thin- and thick-thickness scintillation detectors using the high-resolution line chart phantom in Figure 2b. Figure 9 shows the graph in which the optimal sigma values were identified for each sub-band in the DWT in the simulation. The optimal sigma value from the SSIM results was approximately 2.12, and those from the GM results of the horizontal, vertical, and diagonal directions of the DWT were approximately 1.25, 1.32, and 1.11, respectively.

**Figure 10:**
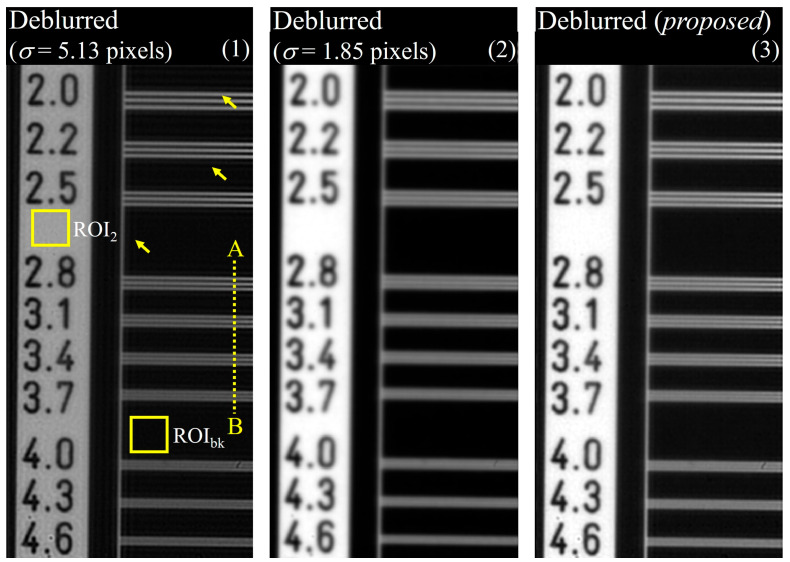
Experimental results using high-resolution line chart phantom: (1) deblurred image with σ = 5.13 pixels, (2) deblurred image with σ = 1.85 pixels, and (3) image obtained with the proposed method. Here, line *AB* is used to measure the profile for comparison the resolution. The yellow arrows are an artifacts that can occur when deblurring is performed using an oversized PSF.

**Figure 11:**
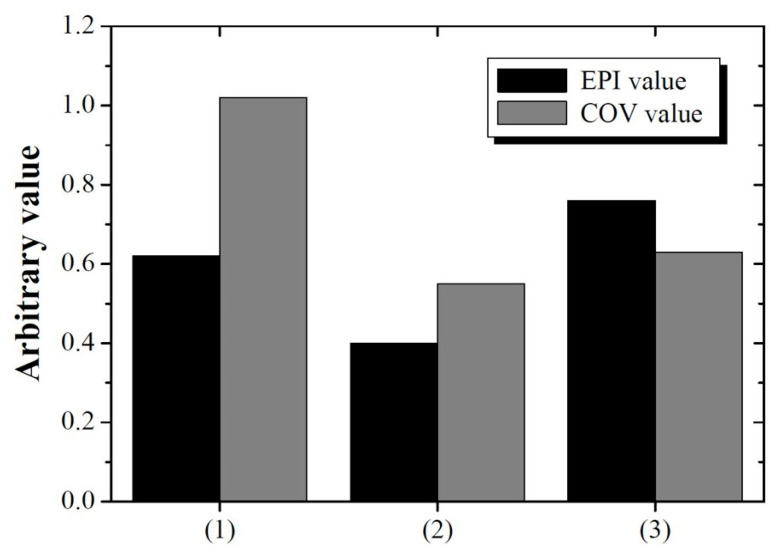
Evaluated EPI and average COV values for (1), (2), and (3) images of Figure 10. Average COVs were calculated in ROI_bk_ and target area (ROI_2_).

Figure 10 shows the experimental results of (1) a deblurred image using σ = 5.13 pixels for detector 2; (2) a deblurred image using the σ = 1.85 pixels for detector 2; and (3) a deblurred image obtained using the proposed method. Image (3) qualitatively verifies that the resolution is significantly improved compared to that of image (2). The yellow arrow shows the result of degradation when image restoration is performed using σ = 5.13 pixels, which shows that image restoration should be performed using an accurate blur kernel. Figure 11 shows the evaluated EPI and average COV results for images (1), (2), and (3). The average COVs were calculated for ROI_bk_ and the target area (ROI2). The EPI values of the three images were approximately 0.62, 0.40, and 0.76, and the COV values were approximately 1.02, 0.55, and 0.63, respectively. These results are similar to the simulation results, demonstrating that the proposed image restoration method is viable for real-world radiographic imaging systems. When using the proposed method (3), we confirmed that EPI and COV were improved by 22.58 and 38.24%, respectively, compared to (1). In addition, the proposed method (3) can improve EPI by nearly twice as much as (2), and the COV difference is about 14.55%. These results mean that the appropriate spatial resolution and noise level can be obtained simultaneously when using the proposed DWT.

**Figure 12:**
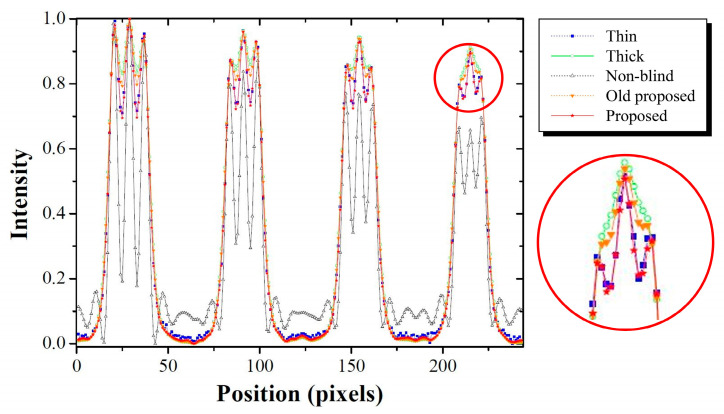
Profiles of images: thin scintillator, thick scintillator, non-blind restored with σ = 5.13 pixels, restored using the image domain approach, and image along the line *AB* in Figure 10 using the proposed method.

This can be observed quantitatively in Figure 12. This result shows the profile of the *AB* line segment, which contains 2.8–3.7 lp/mm. Here, the non-blind category represents the deblurred image with σ = 5.13 pixels in Figure 10. The profile of the restored image obtained using the formerly proposed method in [21] from the thick scintillation image was compared with the profile of the restored image based on the method proposed in this study deriving the optimal parameter for each sub-band in the wavelet domain from the thick scintillation image. The profile obtained by the proposed method was the most similar to that of a thin-thickness scintillation detector in an enlarged profile within the red circle of 3.7 lp/mm. In particular, the proposed method demonstrated sufficient discrimination in comparisons of the profiles with those obtained using the non-blind and formerly proposed category.

The simulation and experimental results demonstrate that the proposed algorithm can optimize the parameters of the deconvolution algorithm, restoring sharpness and minimizing noise amplification in each sub-band. In particular, the experiments were evaluated using the high-resolution line chart phantom for the quality control and quality assessment of real clinical imaging systems, thus confirming the viability of the proposed algorithm in the clinical field. However, this study has several limitations. First, when performing sharpness improvement for each sub-band, artifacts may increase, similar to the deconvolution process. Deconvolution algorithms create ringing artifacts because errors often cause strong oscillations at data discontinuities, such as edges and noise, sometimes manifesting as false edges [37]. These artifacts can distort inverse DWT images; however, if these factors are not sufficiently reflected in the SSIM and GM evaluation values, the proposed algorithm may find it difficult to derive the optimal parameters. To address this issue, a method that reduces ringing artifacts through diffusion filters [37] or PSF frequency analysis [38] can be considered as a solution. Further research is planned for the future. Second, computational costs should also be discussed. Because overall image restoration is performed on a sub-band image with multiresolution, a problem with computational cost may arise. Compared with performing image restoration only in the image domain, the amount of computation can rapidly increase, and there is a limit to applying complex image restoration when using a real-time image, such as C-arm fluoroscopy [39]. The processing time of the proposed algorithm for a 512 × 1024 pixels image was approximately 12 s. To perform real-time image processing, computation time must be improved. Physical computational speed improvement can help solve this problem by improving the GPU and parallel computing performance through hardware development, replacing the current method with an algorithm that can quickly perform image restoration by reducing unnecessary operations and determining a reasonable separation level of DWT. Scatter radiation plays a crucial role in determining the quality of radiographic images. In both medical diagnosis and nondestructive testing, the decrease in image contrast caused by scatter radiation is a significant concern [40]. However, the impact of scatter radiation was not considered in the proposed method. Without calibration using a precise scatter kernel, achieving accurate image restoration becomes challenging. Furthermore, determining the optimal parameter using the proposed method can also prove to be difficult. Additional investigation is required to delineate a novel degradation model and develop an algorithm for determining optimal parameters, considering that the blurring induced by scatter radiation and imaging system characteristics operate through distinct mechanisms. Subsequent evaluation and validation are essential steps in this process. In the future, we plan to research and develop an algorithm that can determine optimal parameters to enable the conversion of the sharpness of a thick scintillator to that of a thin scintillator while considering comprehensive degradation.

## 4. Conclusions

Spatial resolution and noise characteristics are well known for their trade-off relationship in radiographic imaging, aiming to achieve a radiation dose that is as low as can be reasonably achieved with radiation protection. The proposed method consists of a main algorithm for predicting the optimal PSF to restore the resolution of a thick scintillation detector to that of a thin scintillation detector. Among the IQA evaluation methods, SSIM and GM were used to determine the adaptive PSF for each DWT sub-band. In the simulation and experimental results, the quality of the proposed image improved when noise amplification and artifacts were considered. In particular, the resolution of the proposed image was close to that of the thin scintillation detector image, which was in the range of 2.8–3.7 lp/mm in the high-resolution line chart phantom. In conclusion, the proposed method has been demonstrated to effectively improve image performance and is expected to be applicable to various radiological imaging systems.

## Data Availability

The raw data supporting the conclusions of this study will be made available by the authors upon request.
